# The failure and revival of closed microbial ecosystems: evidence from the shifts in microbial and chemical diversities

**DOI:** 10.1093/ismeco/ycag005

**Published:** 2026-01-14

**Authors:** Liang Li, Hao Liu, Jing Ding, Yujia Cai, Peng Zhao, Lu Zhang, Bastian T Steudel, Zimeng Wang, Zheng Chen

**Affiliations:** Department of Health and Environmental Sciences, School of Science, Xi'an Jiaotong-Liverpool University, Suzhou 215123, China; Department of Environmental Science and Engineering, Fudan University, Shanghai 200433, China; Department of Health and Environmental Sciences, School of Science, Xi'an Jiaotong-Liverpool University, Suzhou 215123, China; School of Environmental Science and Engineering, Suzhou University of Science and Technology, Suzhou 215009, China; Department of Health and Environmental Sciences, School of Science, Xi'an Jiaotong-Liverpool University, Suzhou 215123, China; Department of Health and Environmental Sciences, School of Science, Xi'an Jiaotong-Liverpool University, Suzhou 215123, China; Department of Health and Environmental Sciences, School of Science, Xi'an Jiaotong-Liverpool University, Suzhou 215123, China; Institute of Nature Conservation, Polish Academy of Sciences, Mickiewicza 33, Kraków 31-120, Poland; Department of Environmental Science and Engineering, Fudan University, Shanghai 200433, China; Department of Health and Environmental Sciences, School of Science, Xi'an Jiaotong-Liverpool University, Suzhou 215123, China

**Keywords:** chemodiversity, closed microbial ecosystems, microbial diversity, system persistence

## Abstract

Closed microbial ecosystems (CES) are vital models for studying ecosystem stability and resilience, especially in carbon cycling. This study explored algae-bacteria CES using real-time pressure dynamics, 16S rRNA sequencing, and ultrahigh-resolution mass spectrometry. The system exhibited biphasic stability: an initial high-activity phase (Days 1–8) with robust carbon cycling and diverse communities dominated by *Pseudomonas*. Subsequent re-stabilization (Days 31–45) involved a functional shift toward *Brevundimonas* and photoheterotrophic *Porphyrobacter*, coupled with dissolved organic matter (DOM) chemodiversity loss and accumulation of recalcitrant lignin/Carboxyl-rich alicyclic molecules–like compounds. Declining carbon cycling intensity correlated with microbial diversity erosion and DOM simplification, revealing a self-reinforcing feedback loop threatening ecosystem persistence. This work advances frameworks for anticipating tipping points in natural ecosystems under anthropogenic stressors, offering actionable insights for conservation and bioremediation strategies.

## Graphical Abstract

**Graphical Abstract:**
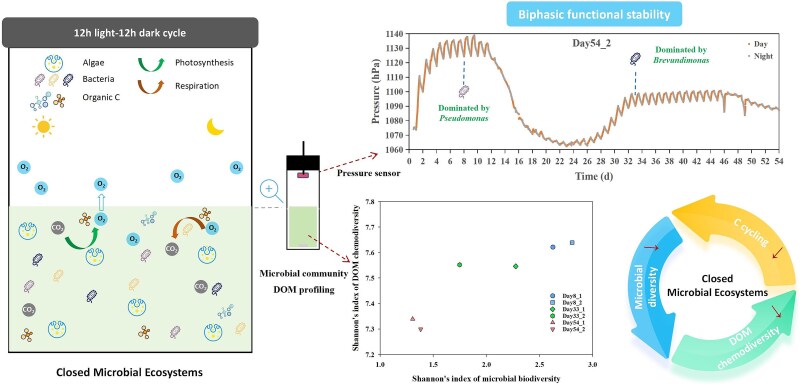

## Introduction

The persistence of closed ecosystems—systems that are materially isolated yet energetically open—has long captivated scientific inquiry, serving as a critical lens to unravel the principles governing Earth’s biosphere and potential extraterrestrial life support systems [1, 2]. While Earth itself functions as a self-sustaining biosphere, conventional ecological research predominantly focuses on open systems, which inherently lack the capacity to mimic feedback mechanisms governing functional stability in isolated environments. This disparity impedes a mechanistic understanding of how ecosystems maintain stability under conditions of severed external nutrient inputs. Microbial biospherics, defined as experimentally tractable, materially closed microbial ecosystems (CES) [3], provide a transformative paradigm to dissect these dynamics through controlled, replicated studies of ecosystem-scale processes.

Recent advances have established CES as powerful model biospheres for probing fundamental ecological dynamics [4, 5]. Typically comprising algae and artificially combined bacterial consortia, CES enable high-resolution tracking of microbial species (e.g. via single-cell imaging) and biogeochemical cycles (e.g. through real-time gas-phase monitoring). For instance, Astacio et al. [6] demonstrated that CES subjected to 12-hour light/12-hour dark cycles self-organize to sustain carbon cycling over months via algae-bacteria symbiosis, with system pressure oscillations mirroring photosynthetic-respiratory coupling. While this prior work established pressure as a powerful proxy for ecosystem-scale function, it primarily captured broad community shifts. An important, unresolved challenge lies in mechanistically linking these macroscopic dynamics to the underlying microbial and molecular drivers. Consequently, important knowledge gaps persist: (i) the specific drivers of CES collapse remain elusive, particularly the role of microbial antagonism in disrupting the critical producer-consumer equilibrium; (ii) the functional coupling between microbial community restructuring and the molecular-level chemodiversity of DOM—a pivotal determinant of ecosystem functionality [7]—has yet to be quantitatively resolved.

A central challenge in this endeavor lies in defining operational thresholds for ecosystem “failure” [3]. Traditional metrics such as species extinction often overlook the gradual metabolic erosion preceding systemic collapse. In CES, gaseous O_2_/CO_2_ dynamics, quantified via pressure fluctuations, provide a powerful, real-time proxy for carbon cycling integrity and algal photosynthetic capacity, which are vital for system survival. However, to our knowledge, no study has simultaneously bridged these macroscopic functional signatures with high-resolution molecular-level DOM transformations and detailed microbial succession patterns. Furthermore, although algicidal bacteria are recognized as potent destabilizers of algal-bacterial consortia [8], their specific impact on the long-term persistence of closed-system—and their potential role in the knowledge gaps outlined above—remains uncharacterized.

To address these interconnected gaps, we employed an integrative biophysical-genomic-chemical framework that distinctively advances beyond prior CES studies. We hypothesize that microbial interactions modulate carbon cycling efficiency, thereby dictating overall system stability and its manifestation in pressure dynamics. To test this, we assembled aquatic algae-bacteria CES units provided with only light, deploying a triad of analytical approaches: real-time pressure monitoring (to quantify O_2_/CO_2_ fluxes), 16S rRNA sequencing (for microbial community profiling), and Fourier Transform Ion Cyclotron Resonance Mass Spectrometry (FT-ICR-MS) (for high-resolution DOM molecular characterization) across a 54-day experiment. This integrated design enables us, for the first time, to directly link ecosystem-scale functional stability and collapse to the coupled trajectories of microbial community succession and DOM molecular evolution.

## Materials and methods

### Preparation of CES establishment

The establishment of CES is illustrated in Fig. S1. Soil samples were collected from a grassland area at Xi’an Jiaotong-Liverpool University and stored in sterile test tubes. A soil microbial suspension was prepared by mixing 5 g of fresh soil with 10 ml of 1.5 g/L glucose solution in a 15 ml tube to provide a readily available carbon source, ensuring the reliable establishment of an active heterotrophic bacterial community in the CES. The mixture was vortexed for 1 min, and pre-incubated for 48 h. After centrifugation at 7000 rpm for 5 min, the supernatant was discarded. The remaining particles were resuspended in an equal volume of Modified-SE (M-SE) medium (composition detailed in Table S1). Cycloheximide (200 μg/ml) and nystatin (20 mg/L) were added to the soil solution to inhibit eukaryotic protein synthesis and fungal growth [9, 10], respectively. The treated solution was wrapped in aluminum foil and incubated at 30°C with shaking at 225 rpm for 48 h. After incubation, 10 samples were taken with 1 ml each, centrifuged at 7000 rpm for 7 min, the supernatant was removed, and the pellet was resuspended in fresh M-SE medium. This washing step was repeated three times to minimize the carryover of cycloheximide and nystatin into the final CES units, thereby reducing their potential direct effects on subsequent algal growth and DOM analysis. The final pellets were combined for CES initiation.

The CES were established in 40 ml glass vials with a liquid medium volume of 20 ml. The model soil-dwelling alga, *Chlamydomonas reinhardtii* (FACHB-265), [11], was sourced from the Freshwater Algae Culture Collection at the Institute of Hydrobiology, National Aquatic Biological Resource Center, China. The alga was cultured in SE medium (see Table S2) at 25°C with a light intensity of 150 μmol·m^−2^·s^−1^ for 5 days. The culture was then transferred to 15 ml sterile tubes and centrifuged at 5000 rpm for 2 minutes. After discarding the supernatant, the pellet was resuspended in ~5 ml of M-SE medium. The algal density in the suspension was then measured via hemocytometry and diluted to 5 × 10^5^ cells/ml for CES initiation.

### Real-time monitoring of headspace pressure

Headspace pressure within the CES units was monitored in real-time as a non-invasive proxy for the net balance between photosynthetic O_2_ production and respiratory CO_2_ consumption, thereby serving as a direct indicator of carbon-cycling activity. The monitoring system (Supplementary Fig. S2) was built around BME280 sensors (Bosch), which measure pressure, temperature, and humidity. The primary ecological metric derived from this system was the diel (24-hour) oscillation in pressure: a pressure increase during the light phase indicated net autotrophy (photosynthesis dominant), while a decrease during the dark phase indicated net heterotrophy (respiration dominant). The amplitude of these oscillations provided a quantitative measure of carbon cycling intensity. Data were recorded at 10-s intervals to resolve these dynamics. A detailed description of the system’s electronic architecture and data acquisition pipeline is provided in the Methods S1.

To isolate pressure changes caused by microbial gas fluxes from those driven by ambient temperature fluctuations, a dedicated calibration procedure was performed. A control vial containing only sterile water was monitored alongside the experimental CES units. The empirical relationship between temperature and pressure, derived from this abiotic control, was used to calibrate all raw pressure data from the biological CES units to an equivalent pressure at a constant temperature of 30°C. All pressure dynamics reported in this study are thus temperature-calibrated values, ensuring that the observed oscillations primarily reflect biological activity (Supplementary Fig. S3).

The airtight integrity of the sealed CES units was rigorously verified. First, the total mass of each vial remained unchanged throughout the 54-day experiment, confirming the absence of measurable gas exchange. Second, upon opening the vials at the endpoint, the pressure sensors immediately returned to and stabilized at ambient atmospheric pressure, confirming both the calibration stability of the sensors and the biological origin of the recorded diel oscillations.

### CES initiation and parameter control

To initiate the CES, 19 ml of M-SE medium was added to a 40 ml glass vial, followed by 0.5 ml each of a soil-extracted bacterial community and *C. reinhardtii* algae solution (algal final concentration: 5 × 10^5^ cells/ml). The vial was then sealed with a sensor-embedded cap, ensuring airtightness using adhesive. The CES units were subjected to a 12-hour light/dark cycle with a light intensity of 150 μmol·m^−2^·s^−1^ and maintained at a constant temperature of 30°C in a water bath.

A total of 12 CES units were assembled and initiated simultaneously under identical conditions. To capture temporal dynamics, destructive sampling was performed at three critical stages: Day 8, two randomly selected CES units were harvested entirely for DOM and microbial community analysis. The remaining CES units continued operation; Day 33, two additional units were destructively sampled, while the remaining units were maintained; Day 54, the final two operational units were sampled, completing the experimental timeline.

At each sampling point, liquid samples from selected CES units were filtered through 0.22-μm polytetrafluoroethylene (PTFE) membranes for downstream analyses. Six units were excluded prior to sampling due to technical failures (sensor malfunctions). A comparative analysis of the high-frequency pressure data confirmed that all 12 units, regardless of their technical fate, exhibited nearly identical functional dynamics during the initial 8-day phase (Supplementary Fig. S4). This confirmed that the biologically retained units (n = 6) were a representative subset of a functionally homogeneous starting population.

### Analysis of DOM molecular composition

For DOM analysis, liquid samples from the CES units were first filtered through 0.22-μm PTFE membranes to remove microbial cells and particulate matter. The resulting filtrate was then applied to solid phase extraction cartridges (Agilent Bond Elut PPL, 1 g) and eluted with methanol. Samples were analyzed using a 15-Tesla Bruker Apex-Ultra FT-ICR MS with electrospray ionization (ESI). Spectra from 128 scans (100-800 Da) were collected. Molecular formulas were categorized into seven compound classes based on H/C and O/C ratios: aliphatic/peptide, lipid, lignin/CRAM-like, carbohydrate, unsaturated hydrocarbon, aromatic, and tannin-like compounds [12], as listed in Table S3. The molecular diversity (chemodiversity) of the DOM pool was calculated as the Shannon diversity index based on mass spectral peak intensities.

### Microbial community analysis

The remaining material from PTFE filtration was used for DNA extraction. Microbial genomic DNA was extracted with the PowerSoil DNA Isolation kit (MO BIO, USA), following the manufacturer’s instructions. The bacterial V3-V4 region of the 16S rRNA genes was amplified using the primers 341F (CCTAYGGGRBGCASCAG) and 806R (GGACTACHVGGGTWTCTAAT), including sample-specific barcodes. The purified amplicons were sequenced on the Illumina MiSeq platform (2 × 250 bp, paired-end) at Novogene Co., Inc., China.

The analysis of 16S rRNA sequences was carried out by QIIME2. Raw pair-end reads were filtered, and the chimeras were removed using the DADA2 plugin. Amplicon sequence variants (ASVs) were generated for the analyses. ASVs with less than 2 reads were omitted to ensure removal of artifacts. Bacterial sequences were classified using SILVA Release 138.1. The *Bray-Curtis* dissimilarities of bacterial communities were calculated based on ASVs. Reads generated in this study were deposited in National Center for Biotechnology Information (NCBI) with accession number PRJNA1227106.

### Statistical analysis between microbial community and DOM composition

Associations between the bacterial ASVs and molecular weight of DOM were examined using Mantel test with the vegan package (version 2.6-4) in R (version 4.5.2) [13]. Mantel test was conducted between the relative abundance of the 20 most abundant ASVs at the genus level (*Bray-Curtis* distance) and the DOM compound series (CHO, CHNO and CHOS) with their molecular weight (from 200 to 600 Da) within the 54-day monitoring periods. The results were plotted as a heatmap using R, with different colors representing Spearman’s rank correlation coefficients.

## Results

### Gas pressure over time

**Figure 1:**
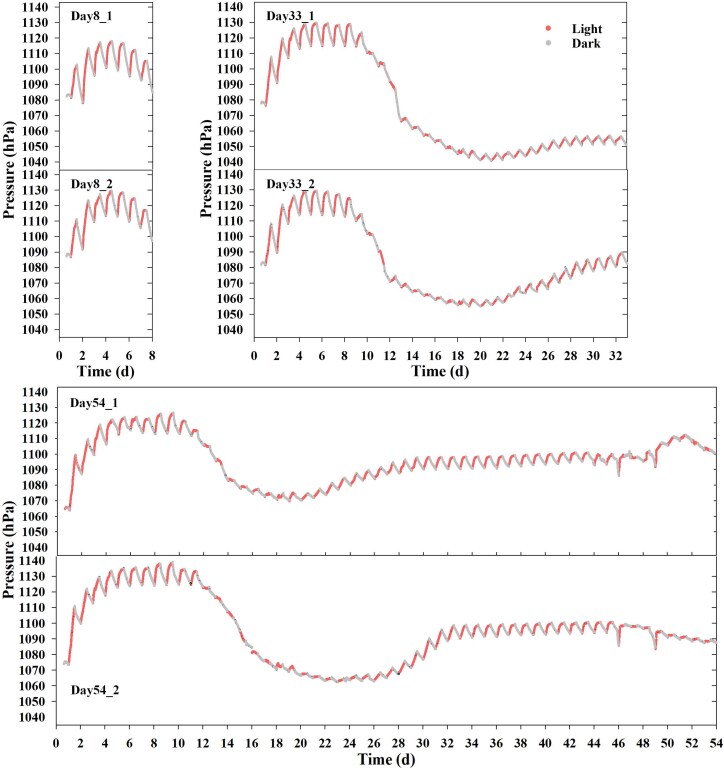
Real-time headspace pressure dynamics in CES units. Pressure oscillations in the CES units (n = 6) subjected to 12 h-12 h light–dark cycles throughout the operation. The red line represents daytime (light phase) and the gray line indicates nighttime (dark phase). Data were collected using a custom-built pressure monitoring system with measurements taken every 10 s. Pressure values are temperature-calibrated.

Given that O_2_ is 30-fold less soluble in water than CO_2_, pressure dynamics were identified as a critical proxy for photosynthetic-respiratory coupling in the CES [6], revealing biphasic stability (Fig. 1). To investigate the driving forces behind these stages, we harvested two CES units each at the end of the 1^st^ stable stage (Day 8) and at the beginning and end of 2^nd^ stable stage (Day 33 and Day 54, respectively).

Pressure variations exhibited regular oscillations in all six CES units during the first eight days of operation (Fig. 1). Within a daily cycle, the pressure amplitude ranged between 10-20 hPa, gradually increasing during 12-hour light phase (photosynthetic O_2_ accumulation) and decreasing during 12-hour dark phase (respiratory CO_2_ release) (Supplementary Fig. S5), confirming active carbon cycling.

Between Days 8 and 33, the CES units experienced a significant pressure decline followed by a slight increase. Initially, daily pressure decreased continuously, with oscillations ceasing. Later, oscillations reappeared, gradually increased in amplitude to 5-8 hPa by Day 33. Extended monitoring to 54 days revealed that pressure oscillations in two CES units remained stable at an amplitude of 6-8 hPa until Day 46, after which the amplitude progressively declined.

To quantitatively assess the balanced and stable nature of the 2^nd^ stable stage (Days 31–45), we analyzed the high-frequency pressure data. First, the net daily pressure change (ΔP_net) over consecutive 24-hour periods was calculated. The mean ΔP_net during this phase was −0.11 hPa (SD = ±0.66 hPa), which was not significantly different from zero (one-sample t-test, *P* = .375), indicating a precise daily balance between net O_2_ production and CO_2_ consumption. Second, the stability of the carbon cycling was evidenced by a low coefficient of variation (CV = 11.56%) in the daily pressure oscillation amplitudes, significantly lower than the CV of 38.70% observed in the first 8 days. These metrics quantitatively define the “balanced” and “stable” state of the CES during this period.

To quantitatively define the biphasic stability and the intensity of carbon cycling, we calculated the daily pressure oscillation amplitude for each CES unit across three key phases: the initial high-activity stable phase 1 (Days 1-8), the re-stabilized phase 2a (Days 31-45), and the final decline phase 2b (Days 46-54). As summarized in Table S4, the mean amplitude (±SD) across all units was 18.68 ± 7.23 hPa during Phase 1, which sharply decreased to 7.22 ± 0.88 hPa during Phase 2a, demonstrating a significant reduction in carbon-cycling intensity. In the subsequent Phase 2b, the amplitude further declined to 4.95 ± 5.15 hPa, underscoring the eventual attenuation of ecosystem function.

This pressure pattern across 54-day periods suggested that the carbon cycling intensity of the microbial ecosystem, as indicated by pressure oscillations, initially exhibited high activity, then sharply weakened, gradually recovered, and finally slowly declined.

### Microbial community succession across stability phases

**Figure 2:**
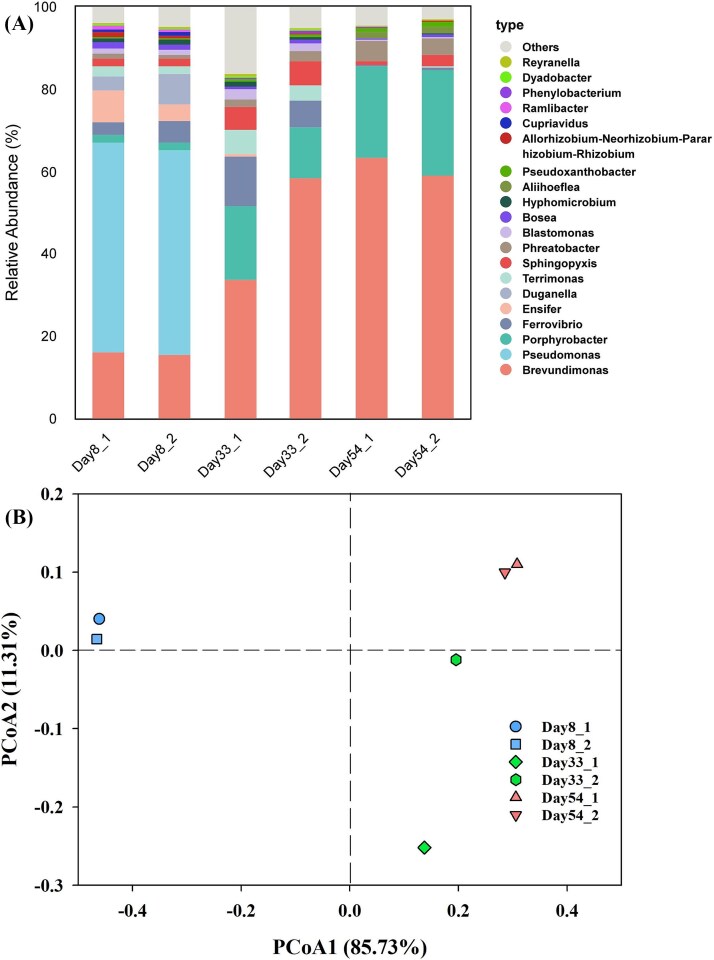
Temporal shifts in bacterial community structure. (A) Relative abundance bar plot of the top 20 most abundant bacterial genera across different time points, based on 16S rRNA gene amplicon sequencing. (B) Principal coordinate analysis (PCoA) of bacterial communities based on *bray-Curtis* dissimilarities. Each point represents an individual replicate (n = 2 per time point). Due to limited replication, confidence ellipses are not shown. PERMANOVA indicates a significant effect of time (R^2^ = 0.777, *P* = .011), though heterogeneity in group dispersion (*P* = .001) suggests cautious interpretation.

After 8 days of operation, the abundance of bacterial genera from highest to lowest were: *Pseudomonas*, *Brevundimonas*, *Ensifer*, *Duganella*, *Ferrovibrio*, *Porphyrobacter*, *Terrimonas*, *Sphingopyxis*, *Blastomonas*, and *Phreatobacter* (Fig. 2A). The sequencing results for reads and ASVs were generally summarized in Table S5, Fig. S6.

Later, we observed a succession in bacterial community composition between 1^st^ and 2^nd^ stable stages. By Day 33, the dominant bacteria had shifted from *Pseudomonas* (49.6 ~ 50.9%) to *Brevundimonas* (34.0 ~ 58.7%), coinciding with the disappearance of *Pseudomonas* and *Duganella*. By Day 54, the relative abundances of *Porphyrobacter* (22.0 ~ 25.5%) and *Phreatobacter* (4.2 ~ 5.1%) continued to rise, while those of *Sphingopyxis*, *Blastomonas*, and *Ferrovibrio* declined, accompanied by the disappearance of *Terrimonas* and *Ensifer*.

Principal Coordinates Analysis (PCoA) results also reflected significant transformation in the microbial community structure over time. Samples from Day 8 exhibited considerable distance from those on Days 33 and 54 (Fig. 2B), indicating substantial differences in community composition between 1^st^ and 2^nd^ stable stages. Conversely, the proximity between samples from Days 33 and 54 suggested greater similarity in community structural composition. Permutational multivariate analysis of variance (PERMANOVA) indicated a significant effect of sampling time on community structure (R^2^ = 0.777, *P* = .011). However, a test for homogeneity of multivariate dispersions revealed significant differences in within-group variation across time points (*P* = .001), suggesting that the PERMANOVA result should be interpreted with caution as it may be partially influenced by heterogeneous variances. This heterogeneity, combined with the limited replication (n = 2 per time point), precludes the calculation of confidence intervals in the PCoA visualization (Fig. 2B). Nonetheless, the consistent directional shifts observed in both replicates at each time point, coupled with the strong PERMANOVA effect size (R^2^ = 0.777), support the biological significance of the community succession patterns.

Clearly, after 54 days of CES operation, the functional stability of the CES underwent shifts in carbon cycling intensity, accompanied by succession in microbial community composition and a decrease in microbial diversity (Table S5).

### DOM molecular evolution

**Figure 3:**
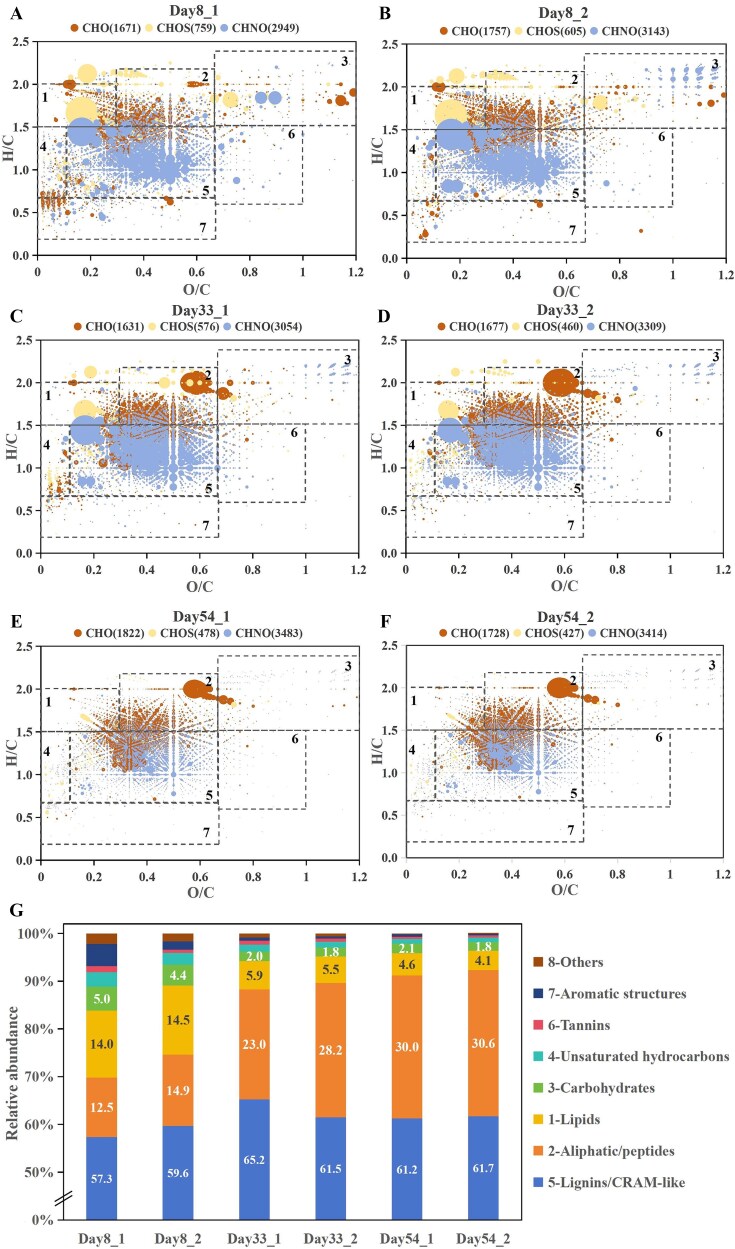
Molecular-level evolution of DOM composition. (A–F) Van Krevelen diagrams illustrating the molecular characteristics of DOM at different time points, as determined by electrospray ionization Fourier transform-ion cyclotron resonance mass spectrometry (ESI FT-ICR-MS). Each data point represents a unique molecular formula, colored by its assigned compound class and sized proportionally to its relative mass spectral intensity. (G) Temporal changes in the relative abundance of DOM containing different elemental compositions in CES. Molecular formulas were categorized into the compound classes shown in the legend based on their H/C and O/C ratios, according to the stoichiometric criteria defined in Table S3.

**Figure 4:**
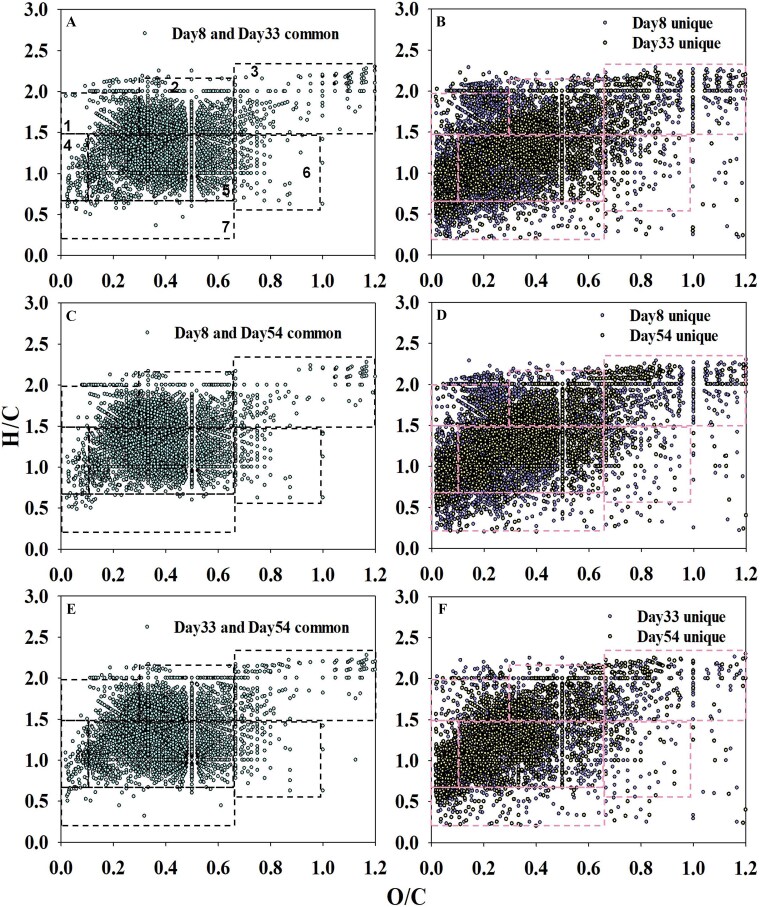
Temporal shifts in the shared and unique molecular composition of DOM. Van Krevelen diagrams illustrating the number of molecular formulae that were common or unique to different sampling days. Panels A, C, E show the shared molecules between time points, while panels B, D, and F show the molecules unique to each time point. Molecular formulas were categorized into the compound classes based on their H/C and O/C ratios, according to the stoichiometric criteria defined in Table S3: 1-lipids; 2-aliphatic/peptides; 3-carbohydrates; 4-unsaturated hydrocarbons; 5-lignin/carboxyl-rich alicyclic molecules (CRAM)–like; 6-tannins; 7-aromatic structures.

As the CES operation progressed, the quantity of CHOS molecules steadily declined from 682 to 518 by Day 33, further dropping to 452 by Day 54. In contrast, CHNO molecules increased from 3046 to 3182 by Day 33, and continued to rise to 3448 by Day 54 (Fig. 3). This pattern indicated a decrease in CHOS molecules with an increase in CHNO molecules in the DOM compositions. Simultaneously, the relative abundance of lignin/CRAM-like molecules in the DOM increased from 58.50 ± 1.63% on Day 8 to 63.36 ± 2.66% by Day 33, before slightly decreasing to 61.49 ± 0.35% by Day 54. Aliphatic/peptide molecules grew from 13.71 ± 1.75% on Day 8 to 25.59 ± 3.66% by Day 33, and further increased to 30.27 ± 0.41% by Day 54 (Table S6). In contrast, the relative abundances of lipids, carbohydrates, unsaturated hydrocarbons, tannins, and aromatic compounds consistently decreased. This pattern indicated a shift in DOM composition toward a greater relative abundance of lignin/CRAM-like compounds and aliphatic/peptide molecules, accompanied by a decrease in the relative abundances of lipids, carbohydrates, unsaturated hydrocarbons, tannins, and aromatic compounds between the 1^st^ and 2^nd^ stable stages, as illustrated by Fig. 4, thereby reducing overall molecular diversity.

### Correlation between microbial community and DOM composition

**Figure 5:**
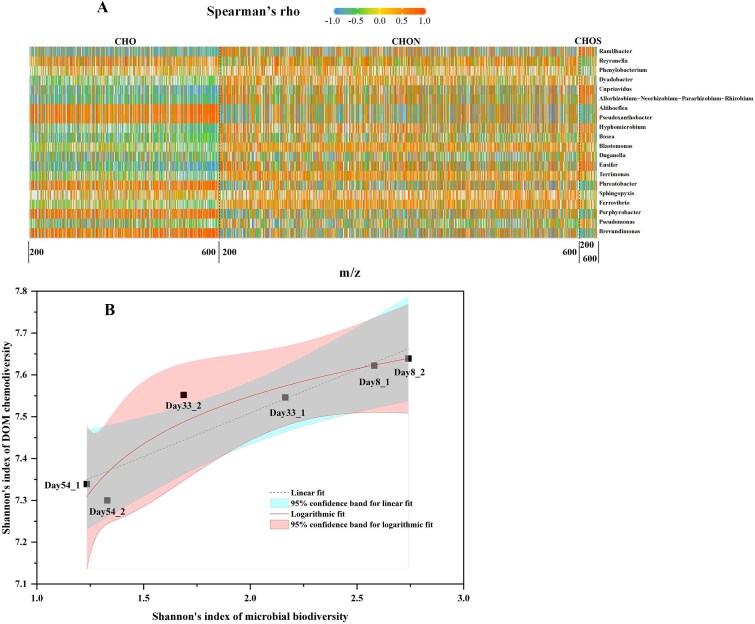
Associations between bacterial community composition and DOM characteristics. (A) Heatmap of Spearman’s rank correlation coefficients (Spearman’s rho) between the relative abundance of the top 20 bacterial ASVs (genus level) and the intensity of DOM compound series across a range of molecular weights (m/z 200–600). Only correlations with |rho| ≥ 0.3 are displayed, highlighting ecologically meaningful relationships. Red indicates positive correlations and blue indicates negative correlations. (B) Scatter plot showing the relationship between the Shannon diversity index of the bacterial communities and the molecular diversity of the DOM pool. The shaded areas represent the 95% confidence bands for each fit. The analysis includes n = 6 biologically independent samples. Logarithmic fitting (red line, R^2^ = 0.9026, *P* = .0304) outperforms linear fitting (black line, R^2^ = 0.8356, *P* = .0108), suggesting that chemical diversity declines more steeply as microbial diversity decreases.

Bacterial communities played a crucial role in DOM compositions, as they can produce, transform, or break down DOM [14, 15]. DOM also acted as an essential energy source for heterotrophic microbial communities [16], sustaining biogeochemical functionalities (C/N cycling). Correlation analysis demonstrated that distinct bacterial groups exhibited diverse metabolic activities in response to various organic compounds, as depicted in Fig. 5A. Notably, almost each genus of bacteria exhibited a uniform correlation with organic molecules containing CHO elements, irrespective of their molecular mass. In contrast, the correlation with organic molecules containing CHON and CHOS elements was more erratic, changing with the molecular mass of these compounds. This pattern of correlation suggested that bacterial communities were significantly associated with the composition of DOM.

Trend analysis of diversity indices revealed that the Shannon diversity index declined for both bacterial communities and DOM components as the CES operated between 1^st^ and 2^nd^ stable stages, as shown in Fig. 5B. This decline in diversity indices indicated a concurrent decrease in the biodiversity of bacterial communities and the chemodiversity of DOM within the CES, while chemical diversity declines more steeply as microbial diversity decreases during 2^nd^ stable stage.

## Discussion

### Microbial community shift

We successfully monitored the microbial community shift during the failure and revival of the gas fluctuations in the CES (i.e. between 1^st^ and 2^nd^ stable stages). During the 1^st^ stable stage, the acclimation period of the microbial community in the CES only took 1 to 3 days, which was more rapid than in a previous study [6]. This rapid acclimation could be attributed to the exclusive inclusion of glucose instead of soil extract in the M-SE medium compared to SE medium. We acknowledge that this alteration in medium composition may have introduced additional confounding factors, such as differences in micronutrient availability, pH buffering capacity, or light attenuation properties, which could have also influenced the early community dynamics. Nonetheless, the provision of a readily available carbon source like glucose likely played a primary role in accelerating the initial growth and metabolic synchronization of the heterotrophic community. *C. reinhardtii* maintained high activity without requiring adaptation to medium change, resulting in significant photosynthetic activity. Similarly, the fresh medium provided abundant nutrients, fostering high growth activity and respiratory metabolism within the bacterial community. The vigorous life processes of both alga and bacteria, characterized by strong photosynthetic and respiratory activities, led to significant fluctuations in oxygen concentrations within the gas phase, thereby causing large pressure oscillation amplitudes. During this nutrient-rich stage, interactions between producers (algae) and consumers (bacteria) were presumably weak. The abundance of resources allowed all species to grow independently without significant inter-species competition. Furthermore, the microbial community structure may have been less dependent on intricate symbiotic relationships or resource-sharing mechanisms. The alga efficiently performed photosynthesis and generated biomass, while consumers found sufficient organic substrates without relying on the metabolic byproducts of other organisms. Sequencing results also revealed a greater diversity in microbial community composition, with *Pseudomonas* dominating the community.

The significant decline in air pressure and the disappearance of pressure oscillations after Day 10 indicated a decrease in the photosynthetic activity of the alga, likely due to enhanced interactions between the alga and the bacterial community. We hypothesize that the presence of *Pseudomonas* and *Duganella*, both known for their algicidal activity, is a plausible mechanism for the functional decline of the CES observed after Day 10. *Pseudomonas*, known for its high metabolic versatility [17], also exhibits non-specific inhibition of diatom growth [18], and supernatants from *Pseudomonas putida* cultures have been shown to have high algicidal activity, suggesting that *Pseudomonas* inhibits algae by secreting extracellular algicidal substances without close contact [19]. Similarly, *Duganella* has been reported as a wide-spectrum algicidal bacterium [20]. Therefore, the documented algicidal potential of *Pseudomonas* and *Duganella* provides a parsimonious explanation for the observed reduction in photosynthesis and further potential system “failure.” The recovery of pressure oscillations in the CES, accompanied by the disappearance of *Pseudomonas* and *Duganella*, supports this hypothesis. While our destructive sampling design cannot establish a precise temporal sequence, the correlation between their presence/absence and the respective functional decline/recovery suggests a potential keystone role for these interactions in modulating CES stability. Future research using conditioned-media assays or direct co-culture is needed to test this hypothesis and establish causality.

During the 2^nd^ stable stage (Days 31-45), pressure oscillations exhibited regular patterns, and our quantitative analysis confirmed this was a period of balanced and stable metabolism (as evidenced by a net daily pressure change of ~0 and a low coefficient of variation in diel amplitude). This indicated that the alga and bacteria had reached a metabolic equilibrium in carbon usage, effectively balancing photosynthesis and respiration. Sequencing results suggested that microbial communities adapted to better decompose and utilize organic carbon provided solely by *C. reinhardtii*. There was a succession from *Pseudomonas* to *Brevundimonas*, along with an increase in the relative abundance of *Porphyrobacter*, *Ferrovibrio*, *Sphingopyxis*, *Terrimonas*, *Blastomonas*, and *Phreatobacter*. *Brevundimonas* is known to be an efficient producer of extracellular polymeric substances [21] and has been reported to degrade numerous organic compounds, including refractory macromolecules [22–24], which likely facilitated the efficient utilization of organic substrates. *Porphyrobacter* is capable of photoheterotrophic growth using organic carbon [25, 26], enabling its abundance to increase significantly within the CES under the specific conditions of 12-h light/dark cycles. During this period, we speculated that the bacterial community formed a symbiotic association with *C. reinhardtii* to maintain photosynthesis and respiration efficiency, facilitating carbon cycling within the system to ensure CES stability and persistence.

As monitoring persisted, the CES experienced a gradual decline in its carbon cycle. During this period (i.e. 2^nd^ stable stage of Days 46–54), a noticeable reduction in the amplitude of pressure oscillation was observed, indicating diminished intensity in both photosynthesis and respiration. This decline may be attributed to the accumulation of an unavailable pool of essential resources (e.g. carbon) within the CES. Sequencing results confirmed a further decrease in microbial community diversity, with the disappearance of *Terrimonas* and a shift in community composition towards *Brevundimonas*, *Porphyrobacter*, and *Phreatobacter*. Biodiversity loss has been identified as a major driver of declining multifunctionality in agricultural ecosystems [27, 28], and the shift in the microbial community within the CES was anticipated to affect the metabolic capacity for processing organic carbon, leading to the accumulation of recalcitrant carbon and a subsequent decline in carbon cycling efficiency. The change in community composition suggested a reallocation of functional roles among microbes, potentially reducing the overall efficiency of carbon transformation and cycling within the CES.

### Decline in DOM chemodiversity

DOM chemodiversity has been reported to be governed by microbial community assembly in inland waters, rather than the reverse [29]. We successfully tracked the shift in DOM compositions during the failure and revival of the CES (i.e. between 1^st^ and 2^nd^ stable stages). DOM characterizations revealed that, compared to 1^st^ stable stage, DOM molecules increasingly clustered around lignin/CRAM-like and aliphatic/peptide types, with decreased molecular diversity during the 2^nd^ stable stage. The amplitude of pressure oscillations during 2^nd^ stable stage was diminished compared to the 1^st^ stable stage, likely due to the increased presence of less utilizable organic carbon. Notably, DOM chemodiversity declined faster than microbial diversity by Day 54, implying preferential depletion of labile substrates. This was further evidenced by a higher proportion of recalcitrant lignin/CRAM-like molecules [30] and more molecules with negative nominal oxidation state of carbon (NOSC) values [31] (Figs. S7, S8). Kolmogorov–Smirnov tests confirmed highly significant differences between all pairs of time points (Day 8 vs. Day 33: D = 0.0526, *P <* .001; Day 8 vs. Day 54: D = 0.0473, *P* < .001; Day 33 vs. Day 54: D = 0.0459, *P <* .001). This statistical evidence strongly supports the transformation of the DOM pool toward more reduced, recalcitrant compounds. During the later phase of 2^nd^ stable stage (Days 46–54), DOM molecules were further clustered around lignin/CRAM-like and aliphatic/peptide types, exceeding 90% in proportion.

While our non-targeted FT-ICR-MS data cannot elucidate the specific metabolic pathways responsible, the observed molecular-level shifts in the DOM pool are consistent with the well-established microbial processing of organic matter, wherein labile compounds are preferentially utilized, leading to the relative enrichment of more recalcitrant molecules such as lignin/CRAM-like compounds.

### Correlation between decline of microbial diversity and DOM chemodiversity

Considering the decrease in microbial community composition and DOM molecular diversity, we predicted a continuous decline in the intensity of CES carbon cycling and a subsequent decrease in system functional stability and persistence, potentially leading to system “failure.” Interestingly, the decline of the microbial community was not linearly correlated to the decline of DOM chemodiversity. We hence suggest that those bacteria which disappeared during the experiment were more important to maintain DOM chemodiversity than those survived. Such shifts in bacterial communities can be studied in more details in CES model systems. However, it cannot be ruled out that microbial succession, such as the disappearance of *Pseudomonas* and *Duganella*, was the potential driver of the second revival of the CES.

### Experimental design boundaries and analytical considerations

Our study was designed to investigate the stability dynamics of a defined closed algae-bacteria ecosystem. Two foundational choices—the initial glucose amendment and fungal suppression—were essential to establish this tractable model system but inevitably shaped its trajectory. The glucose pulse ensured reliable establishment of an active heterotrophic community, enabling observation of the biphasic stability pattern that is a central finding of this work. Similarly, antifungal pre-treatment with cycloheximide and nystatin, followed by rigorous washing, was necessary to maintain a reproducible algae-bacteria system over the 54-day experiment by preventing fungal overgrowth. We acknowledge that this pre-selection fundamentally shaped the community assembly, and therefore the observed succession from *Pseudomonas* to *Brevundimonas* must be interpreted as properties of this fungal-free bacterial consortium. The thorough washing procedure and absence of these pharmaceuticals in our FT-ICR-MS spectra confirm that the reported DOM dynamics are genuine products of microbial activity rather than analytical artifacts.

Consequently, we explicitly define the scope of this work as characterizing the functional stability of a defined type of CES—one initiated with labile carbon and maintained with a bacterial-dominated microbiome. While this design precludes insights into fungal roles or systems without initial carbon amendments, it provided the controlled framework necessary to mechanistically link community succession, DOM molecular evolution, and ecosystem function for the first time.

We also note limitations in our statistical inferences. The combination of limited replication (n = 2 per time point) and significant heterogeneity in group dispersion, as revealed by our beta-dispersion analysis (*P* = .0014), constrains the robustness of multivariate statistical comparisons. However, the consistent directional patterns observed across independent replicates, coupled with converging evidence from high-resolution DOM chemistry and system-level functional metrics, provide compelling support for the ecological transitions described. Future studies can build upon this baseline to dissect individual factors using fungicide-free systems or varying resource conditions.

The CES has proven to be an excellent model for investigating microbial interactions, particularly in understanding ecosystem persistence and system failure. This system offers critical insights into the stability and resilience of ecosystems, especially in the context of microbial community dynamics and carbon cycling. The adaptation of microbial communities within the CES, particularly through the exclusion of algicidal genera such as *Pseudomonas* and *Duganella*, has been successfully monitored. This adaptation highlights the importance of complex symbiotic relationships and resource-sharing mechanisms in maintaining ecological functional stability (e.g. carbon cycling) and resilience. These findings open avenues for further exploration into how microbial interactions influence ecosystem persistence under varying environmental conditions.

The intricate interplay between microbial biodiversity and DOM chemodiversity is crucial for ecosystem functional stability and resilience. Both microbial diversity and DOM composition significantly contribute to the system’s capacity to withstand disturbances and maintain functionality over time. In this study, the observed decline in both microbial biodiversity and DOM chemodiversity within the CES corresponded with a decrease in functional stability, as indicated by the reduced intensity of carbon cycling. This relationship underscores the importance of maintaining biodiversity and chemical diversity in sustaining ecosystem functionality.

Understanding the linkages between microbial diversity, DOM composition, and their influence on carbon cycling within CES is vital for developing predictive models that forecast the future state and functional dynamics of ecosystems. Such models could help anticipate how ecosystems respond to environmental changes, such as nutrient availability, temperature fluctuations, and anthropogenic disturbances. By elucidating the mechanisms underlying ecosystem stability and resilience, this research contributes to the broader goal of preserving and restoring ecosystems in the face of global environmental challenges.

## Supplementary Material

Supplementary_information_ycag005

## Data Availability

Raw sequences reads generated in this study were deposited in NCBI under BioProject accession number PRJNA1227106. Relevant data are available within the paper and supplementary files.
