# Supplementary material for: The failure and revival of closed microbial ecosystems: evidence from the shifts in microbial and chemical diversities
Source: ISME Commun. 2026 Jan 14;6(1):ycag005. doi: 10.1093/ismeco/ycag005 (PMC12887307; doi:10.1093/ismeco/ycag005)
Supplement: Supplementary_information_ycag005 [file supplementary_information_ycag005.docx]

**Supporting Information for**

**The Failure and Revival of Closed Microbial Ecosystems: Evidence from the Shifts in Microbial and Chemical Diversities**

Liang Li^1,2^, Hao Liu^1^, Jing Ding^3^, Yujia Cai^1^, Peng Zhao^1^, Lu Zhang^1^, Bastian T. Steudel^4^, Zimeng Wang^2^, and Zheng Chen^1,^*

^1^Department of Health and Environmental Sciences, School of Science, Xi'an Jiaotong-Liverpool University, Suzhou 215123, China

^2^Department of Environmental Science and Engineering, Fudan University, Shanghai 200433, China

^3^School of Environmental Science and Engineering, Suzhou University of Science and Technology, Suzhou 215009, China

^4^Institute of Nature Conservation, Polish Academy of Sciences, Mickiewicza 33, Kraków, 31-120, Poland

*Correspondence

Zheng Chen

**Email:**  [zheng.chen@x](mailto:xxxxx@xxxx.xxx)jtlu.edu.cn

**Methods S1: Detailed description of the pressure monitoring system**
The custom pressure monitoring system comprised the following components and workflow.

**Sensors**: BME280 sensors (Bosch) were used for their high precision in measuring atmospheric pressure.

**Microcontroller unit (MCU):** A NodeMCU board, featuring an ESP8266 Wi-Fi module, served as the central processing and communication unit.

**Communication:** Sensors communicated with the NodeMCU via the Inter-Integrated Circuit (I2C) protocol. An I2C hub and a 1-to-8 multiplexer were used to manage multiple sensors simultaneously.

**Data flow:** The NodeMCU, programmed using the Arduino IDE, acted as an Message Queuing Telemetry Transport (MQTT) client. It published raw sensor data at set intervals to an MQTT broker (EMQX) running on a local computer.

**Data storage:** The broker relayed the data via a webhook to a local web service built using the Flask Python package. This service parsed the incoming messages and stored the time-stamped pressure, temperature, and humidity readings in a Structured Query Language (SQL) database for subsequent analysis and visualization.

**Fig. S1** Schematic diagram of closed microbial ecosystems (CES) establishment (Step I-collection of topsoil samples; Step II-sample pre-treatment; Step III-extracting heterotrophic microorganisms; Step IV-culturing autotrophic microorganisms; Step V-light intensity control; Step VI-system stirring control; Step VII-temperature control; Step VIII-monitoring system pressure).

**Fig. S2** Schematic diagram of pressure monitoring system.

**Fig. S3. Demonstration of temperature calibration for pressure data.** Data from a representative CES unit (Day8_1) is shown. (a) The raw pressure signal contains variations due to both biological activity and ambient temperature changes. (b) The final, temperature-calibrated pressure trace used in all analyses. The calibration process effectively removes the temperature-driven noise, revealing the clear diel oscillations attributable to microbial photosynthesis and respiration.

**Fig. S4. Functional trajectories of all twelve CES replicate units during the initial stable phase.** Headspace pressure data for each individual unit (a-l) over the first 8 days. Units are grouped by their final status: Retained units (a-f), used for destructive sampling; Excluded units (g-l), lost due to technical failures. Note that excluded units g and h produced complete and normal traces for the first 8 days before sensor failure. Excluded units i, j, k, and l experienced a temporary data gap (days ~3-4) due to a loose power connection, but their pressure oscillations before and after the gap were continuous with the pattern observed in all other units.

**Fig. S5** The intensity of photosynthesis/respiration (µmol ΔO_2_ per day) for all six CES. We assume respiratory and photosynthetic quotients of 1 and pH 6.5; the rate of respiration is constant during both the light and dark phases for one day. And pressure varies with [O_2_]_g_ linearly (slope=10206.5 hPa/mol, r^2^=0.994).

**Fig. S6. Rarefaction curves of Shannon diversity index.** The curves depict the Shannon index value in relation to sequencing depth for each individual sample. The approach of all curves to a stable plateau indicates that the sequencing depth performed in this study was sufficient to reliably capture the microbial diversity within each sample.

**Fig. S7** Dot plots of Nominal Oxidation State of Carbon (NOSC) and Double Bond Equivalents minus Oxygen atoms (DBE-O)/C of dissolved organic matters (DOM) from CES samples under different monitoring periods. Ⅰ: NOSC<0, (DBE-O)/C>0, unsaturated and reduced compounds; Ⅱ: NOSC>0, (DBE-O)/C>0, unsaturated and oxidized compounds; Ⅲ: NOSC<0, (DBE-O)/C<0, saturated and reduced compounds; Ⅳ: NOSC>0, (DBE-O)/C<0, saturated and oxidized compounds.


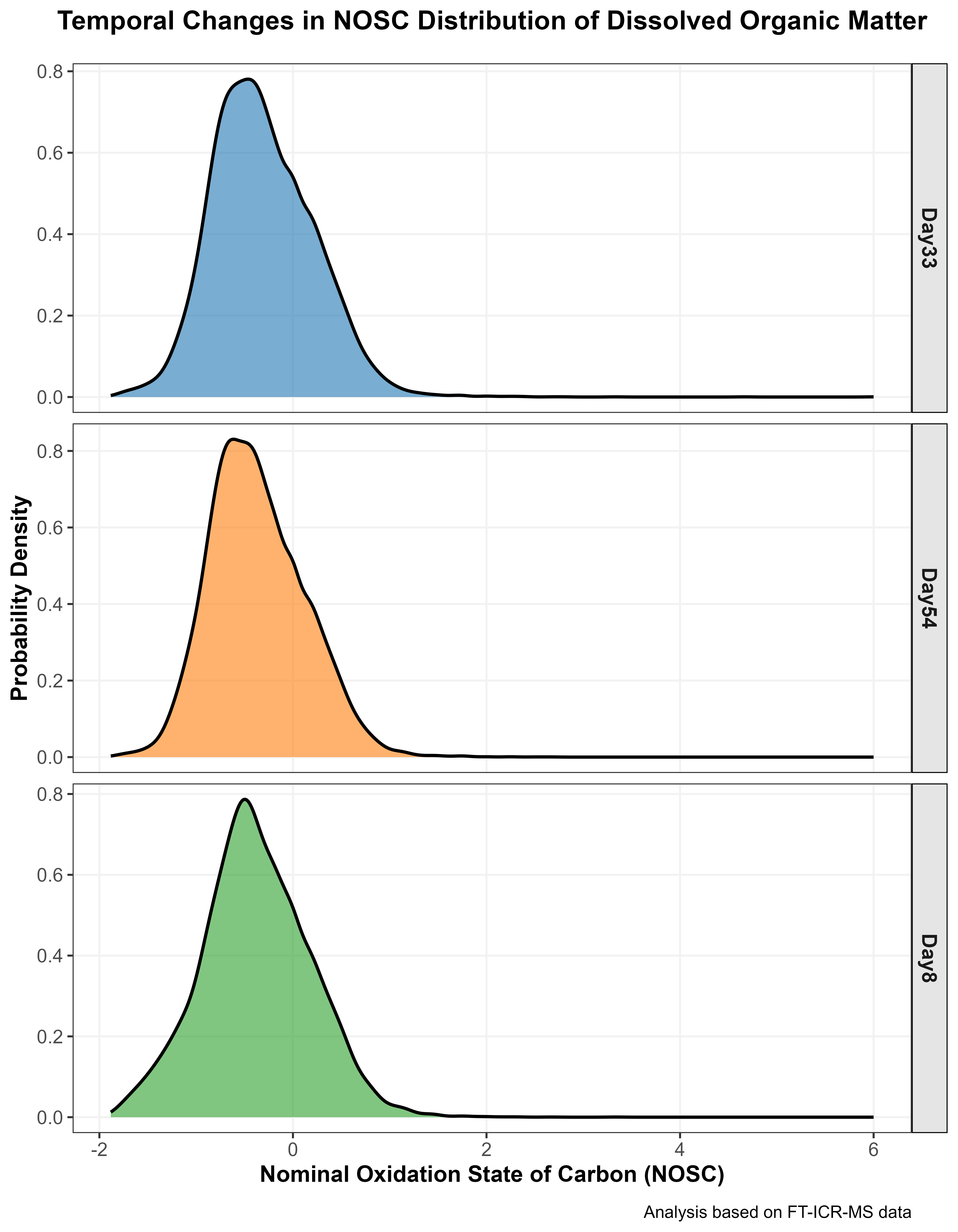


**Fig. S8. Probability density distributions of the NOSC for DOM molecules across time points.** Kolmogorov-Smirnov tests confirm highly significant differences between all time point pairs (all p < 0.001), with D-statistics of 0.0526 (Day 8 vs. Day 33), 0.0473 (Day 8 vs. Day 54), and 0.0459 (Day 33 vs. Day 54).

**Table S1 Modified-SE medium**

| **#** | **Composition** | **Quantity** | **Stock concentration** |
| --- | --- | --- | --- |
| 1 | NaNO_3_ | 1 mL/L | 25 g/100ml dH_2_O |
| 2 | K_2_HPO_4_ | 1 mL/L | 7.5 g/100ml dH_2_O |
| 3 | MgSO_4_∙7H_2_O | 1 mL/L | 7.5 g/100ml dH_2_O |
| 4 | CaCl_2_∙2H_2_O | 1 mL/L | 2.5 g/100ml dH_2_O |
| 5 | KH_2_PO_4_ | 1 mL/L | 17.5 g/100ml dH_2_O |
| 6 | NaCl | 1 mL/L | 2.5 g/100ml dH_2_O |
| 7 | FeCl_3_∙6H_2_O | 1 mL/L | 0.5 g/100ml dH_2_O |
| 8 | EDTA-Fe* | 1 mL/L |  |
| 9 | A5** | 1 mL/L |  |
| 10 | Glucose | 1 mL/L | 0.833 mol/L |

**EDTA-Fe***

1N HCl: take 4.1 ml concentrated hydrochloric acid and dilute it to 50ml with distilled water.

0.1N EDTA-Na_2_: weigh 0.9306g and dissolve it into 50ml distilled water.

0.901g FeCl_3_∙6H_2_O is weighed and dissolved into 10ml of 1N HCl, then mixed with 10ml of 0.1N EDTA-Na_2_, further diluted to 1000ml with distilled water.

**A5****

| **Composition** | **Concentration** |
| --- | --- |
| H_3_BO_3_ | 2.86 g/L dH_2_O |
| MnCl_2_∙4H_2_O | 1.86 g/L dH_2_O |
| ZnSO_4_∙7H_2_O | 0.22 g/L dH_2_O |
| Na_2_MoO_4_∙2H_2_O | 0.39 g/L dH_2_O |
| CuSO_4_∙5H_2_O | 0.08 g/L dH_2_O |
| Co(NO_3_)_2_∙6H_2_O | 0.05 g/L dH_2_O |

**Table S2 SE medium**

| **#** | **Composition** | **Quantity** | **Stock concentration** |
| --- | --- | --- | --- |
| 1 | NaNO_3_ | 1 mL/L | 25 g/100ml dH_2_O |
| 2 | K_2_HPO_4_ | 1 mL/L | 7.5 g/100ml dH_2_O |
| 3 | MgSO_4_∙7H_2_O | 1 mL/L | 7.5 g/100ml dH_2_O |
| 4 | CaCl_2_∙2H_2_O | 1 mL/L | 2.5 g/100ml dH_2_O |
| 5 | KH_2_PO_4_ | 1 mL/L | 17.5 g/100ml dH_2_O |
| 6 | NaCl | 1 mL/L | 2.5 g/100ml dH_2_O |
| 7 | FeCl_3_∙6H_2_O | 1 mL/L | 0.5 g/100ml dH_2_O |
| 8 | EDTA-Fe* | 1 mL/L |  |
| 9 | A5** | 1 mL/L |  |
| 10 | Soil extract*** | 40 mL/L |  |

**EDTA-Fe***

1N HCl: take 4.1 ml concentrated hydrochloric acid and dilute it to 50ml with distilled water.

0.1N EDTA-Na_2_: weigh 0.9306g and dissolve it into 50ml distilled water.

0.901g FeCl_3_∙6H_2_O is weighed and dissolved into 10ml of 1N HCl, then mixed with 10ml of 0.1N EDTA-Na_2_, further diluted to 1000ml with distilled water.

**A5****

| **Composition** | **Concentration** |
| --- | --- |
| H_3_BO_3_ | 2.86 g/L dH_2_O |
| MnCl_2_∙4H_2_O | 1.86 g/L dH_2_O |
| ZnSO_4_∙7H_2_O | 0.22 g/L dH_2_O |
| Na_2_MoO_4_∙2H_2_O | 0.39 g/L dH_2_O |
| CuSO_4_∙5H_2_O | 0.08 g/L dH_2_O |
| Co(NO_3_)_2_∙6H_2_O | 0.05 g/L dH_2_O |

**Soil extract *****

Preparation method of soil extract: take 200g of unfertilized garden soil and place it in a beaker or triangle bottle, add 1000 ml of distilled water, seal the bottle with a breathable plug, heat it in boiling water in a water bath for 3 hours, cool it, and precipitate it for 24 hours. This process is carried out for 3 consecutive times, and then filter, take the supernatant, sterilize it in an autoclave and store it in a refrigerator at 4℃ for later use.

**Table S3 Stoichiometric ranges of the classifications for Van-Krevelen diagrams**

| **Regions** | **Class** | **H/C** | **O/C** |
| --- | --- | --- | --- |
| 1 | Lipids | 1.5 < H/C ≤ 2.0 | 0 ≤ O/C ≤ 0.3 |
| 2 | Aliphatic/peptides | 1.5 < H/C ≤ 2.2 | 0.3 < O/C ≤ 0.67 |
| 3 | Carbohydrates | 1.5 < H/C ≤ 2.4 | 0.67 ≤ O/C < 1.2 |
| 4 | Unsaturated hydrocarbons | 0.7 < H/C ≤ 1.5 | O/C < 0.1 |
| 5 | Lignin/carboxyl-rich alicyclic molecules (CRAM)-like | 0.7 < H/C ≤ 1.5 | 0.1 < O/C < 0.67 |
| 6 | Tannins | 0.6 ≤ H/C ≤ 1.5 | 0.67 < O/C < 1.0 |
| 7 | Aromatic structures | 0.2 < H/C ≤ 0.7 | O/C ≤ 0.67 |

**Table S4. Daily pressure oscillation amplitudes (hPa) for individual CES units across different functional phases.**

| **CES Unit** | **Phase 1 (Days 1-8)**  **Mean±SD** | **Phase 2a (Days 31-45)**  **Mean±SD** | **Phase 2b (Days 46-54)**  **Mean±SD** |
| --- | --- | --- | --- |
| Day8_1 | 22.59 ± 5.57 | - | - |
| Day8_2 | 20.48 ± 4.65 | - | - |
| Day33_1 | 18.79 ± 7.28 | - | - |
| Day33_2 | 18.62 ± 7.14 | - | - |
| Day54_1 | 17.34 ± 9.46 | 7.12 ± 0.45 | 5.78 ± 6.07 |
| Day54_2 | 17.60 ± 8.45 | 7.33 ± 1.19 | 4.12 ± 4.46 |
| All units | 18.68 ± 7.23 | 7.22 ± 0.88 | 4.95 ± 5.15 |

Note: SD, standard deviation. Phases are defined based on the system's functional state derived from pressure dynamics. Data are temperature-calibrated values.

**Table S5 The summarized sequencing results**

| **CES unit** | **Reads** | **Amplicon Sequence Variants (ASVs)** | **Shannon** |
| --- | --- | --- | --- |
| Day8_1 | 600925 | 68 | 2.581 |
| Day8_2 | 67823 | 75 | 2.741 |
| Day33_1 | 106280 | 58 | 2.164 |
| Day33_2 | 45733 | 55 | 1.688 |
| Day54_1 | 94162 | 59 | 1.234 |
| Day54_2 | 88341 | 58 | 1.330 |

**Table S6 Relative abundance (%, Mean ± SD, n=2) of major DOM compound classes across time points.**

| **Class** | **Relative abundance (%, Mean ± SD, n=2)** | | |
| --- | --- | --- | --- |
|  | **Day8** | **Day33** | **Day54** |
| Lipids | 14.26 ± 0.35 | 5.75 ± 0.28 | 4.34 ± 0.38 |
| Aliphatic/peptides | 13.71 ± 1.75 | 25.59 ± 3.66 | 30.27 ± 0.41 |
| Carbohydrates | 4.69 ± 0.47 | 1.94 ± 0.15 | 1.93 ± 0.18 |
| Unsaturated hydrocarbons | 2.75 ± 0.42 | 1.33 ± 0.14 | 0.97 ± 0.018 |
| Lignin/carboxyl-rich alicyclic molecules (CRAM)-like | 58.50 ± 1.63 | 63.36 ± 2.66 | 61.49 ± 0.35 |
| Tannins | 0.98 ± 0.42 | 0.71 ± 0.11 | 0.44 ± 0.041 |
| Aromatic structures | 3.20 ± 2.06 | 0.59 ± 0.18 | 0.41 ± 0.13 |
